# Management Protocol for Uncomplicated Peritonsillar Abscess: Standardizing Care and Improving Emergency Medicine Resident Confidence

**DOI:** 10.7759/cureus.53223

**Published:** 2024-01-30

**Authors:** John Casey, Scott Keidel, Marissa Kudo, Miriam Chan

**Affiliations:** 1 Department of Emergency Medicine, OhioHealth Doctors Hospital, Columbus, USA; 2 Department of Otolaryngology, OhioHealth Doctors Hospital, Columbus, USA; 3 Department of Otolaryngology, Rutgers New Jersey Medical School, Newark, USA; 4 Department of Medical Education, OhioHealth Doctors Hospital, Columbus, USA

**Keywords:** medical education, emergency medicine, tretment protocol, quality improvement projects, otolaryngology emergency, peritonsillar phlegmon, emergency medicine resident

## Abstract

Introduction: A peritonsillar abscess (PTA) is a frequent reason for a visit to the emergency department. As there are no current published guidelines for medical versus surgical management, attending physicians vary among management tendencies, generating uncertainty among resident physicians. This project established a standard of care for managing patients with PTA and provided clear management guidelines to the emergency department, in collaboration with the otolaryngology department, at a community academic hospital.

Methods: Pre- and post-interventional, anonymous surveys were given to assess resident physician confidence in the management of PTA. A proposed management protocol was developed based on existing literature and approved by both the emergency medicine (EM) and otolaryngology (ENT) departments. The protocol was then disseminated during in-person didactic sessions to EM residents and ENT residents for use over a four-month interventional period.

Results: The mean confidence level for all residents increased significantly after the implementation of the protocol (p<0.001). The increase in confidence level for “antibiotic selection for PTA” (p=0.72) and “inpatient PTA management” (p=0.20) was not statistically significant for the post-graduate year (PGY) 3 and 4 residents. The increase in confidence level was higher overall for PGY 1 and 2 residents (95% CI 2.25 ± 1.09, p<0.001) than for PGY 3 and 4 residents (95% CI 1.73 ± 1.09, p=0.003).

Conclusion: The implementation of a standardized protocol for the management of PTA proved to be an effective tool in assisting residents and improving their confidence. This study highlights the importance of establishing guidelines in clinical practice to promote consistent and evidence-based management strategies for PTA. By providing clear guidelines, this protocol enhances communication among healthcare providers and contributes to the delivery of high-quality care to patients with PTA.

## Introduction

Peritonsillar abscess (PTA) is a frequently encountered complication of tonsillitis in the emergency department, yet there is a lack of standardized guidelines for its medical and surgical management [1]. This absence of standardized protocols leads to variability in PTA management, resulting in uncertainty among healthcare providers regarding the appropriate approach. Attending physicians often employ different treatment strategies based on their individual experience, further contributing to this uncertainty. This subjectivity poses challenges for residents in determining when medical management alone is sufficient or when additional surgical intervention is necessary. As emergency medicine (EM) residents and faculty members are often the first to assess and treat PTA patients, we proposed the implementation of a treatment algorithm specifically designed for EM residents. Our aim was to establish a comprehensive protocol that enhanced the confidence and decision-making abilities of EM residents, ultimately leading to improved patient care.

Background

PTAs have an incidence of around 1/10,000 individuals and have a predilection for adolescents and younger adults; however, they can occur at any age [1,2]. They occur when a pocket of pus forms in the peritonsillar space, the area between the tonsillar capsule and the superior constrictor muscle, more frequently occurring unilaterally but can be found bilaterally as well [1]. The diagnosis of PTA can often be made clinically, based on history and physical examination alone. Patients will classically exhibit signs of trismus, uvular deviation, muffled voice (“hot potato” voice), and soft palate edema, with associated symptoms such as odynophagia and increasing unilateral neck pain. Further radiologic investigation may be obtained, often using a CT neck with contrast, to verify the presence and size of the abscess [1].

At our institution, patients will routinely undergo further workups with a CT scan of the neck. CT results are frequently interpreted as either phlegmonous cellulitis or a measurable abscess of the peritonsillar space. When an abscess is observed, a decision must be made to treat it with medical management alone or with the addition of surgical management using incision and drainage (I&D). There are no universally accepted guidelines based on abscess size, and treatment protocols vary by institution. Our protocol followed a proposed set of guidelines by Battaglia et al. [3] to implement a standardized management strategy for uncomplicated PTA and to enhance resident confidence in management.

Size selection

The study by Urban et al. [4] supports the treatment of abscess sizes <2 cm using medical management alone. Due to resident physician and training availability, our institution has historically attempted drainage of much smaller abscesses, and we anticipated a difficult transition to endorse solely medical management for all PTAs <2 cm. To aid in this transition in management, the size cut-off for this protocol was 1.7 cm, based on the aforementioned study by Urban et al. [4], where 1.69 cm was the average size of an abscess treated successfully with medical management alone.

Antibiotic selection

The most common pathogen revealed in culture is Group A beta-hemolytic streptococcus. However, these infections are usually polymicrobial, requiring coverage for gram-positives, gram-negatives, and anaerobes [1,2]. Amoxicillin-clavulanic acid, or a combination of clindamycin and ceftriaxone, may be used. Clindamycin is primarily bacteriostatic versus the bactericidal properties of amoxicillin-clavulanic acid. Multiple studies have demonstrated increased resistance to clindamycin. If clindamycin is to be used, it should be combined with ceftriaxone [4].

Steroids

One dose of dexamethasone 10 mg IV has been shown to improve pain, trismus, dysphagia, body temperature, and hours of hospitalization [5,6].

## Materials and methods

Protocol

A protocol for the management of uncomplicated PTA was adopted from that used by Battaglia et al. [3]. The protocol and treatment algorithm were approved by both ENT and EM attending physicians at OhioHealth Doctors Hospital in Columbus, Ohio. The patient population consisted of those aged ≥18 years, immunocompetent, with the presence of an uncomplicated phlegmon or abscess of the peritonsillar space (without extension into additional neck spaces) confirmed by CT scan. Exclusion criteria consisted of age <18 years, infection involving additional neck spaces, current chemotherapy treatment, current immunosuppressive medication, active malignancy, or uncontrolled diabetes (A1c ≥8 or blood glucose ≥180 ml/dL).

Upon assessment of a patient with clinical suspicion of PTA meeting inclusion criteria in the emergency department, all patients received IV fluids: dexamethasone 10 mg IV, ceftriaxone 2 g IV, and clindamycin 600 mg IV. Ampicillin-sulbactam was an acceptable alternative for patients with allergies, precluding ceftriaxone or clindamycin use. A CT of the neck with IV contrast was recommended to preclude the involvement of multiple neck spaces.

**Figure 1:**
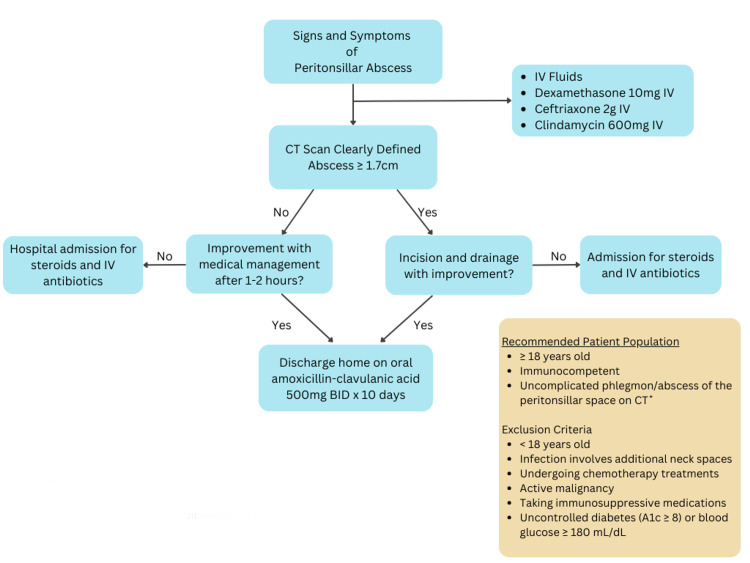
PTA treatment algorithm IV: intravenous, CT: computerized tomography, BID: twice daily, PTA: peritonsillar abscess, *Uncomplicated: infection does not involve additional neck spaces

If a CT scan revealed an isolated peritonsillar phlegmon of any size or an abscess size <1.7cm, observation for one to two hours after initial medical management was recommended. If improvement during that time frame was observed, the patient was discharged on oral antibiotics, with the suggested use of amoxicillin-clavulanic acid 500 mg orally twice daily for 10 days. If no clinical improvement was observed during the emergency department observation period, as assessed by the emergency department attending physician, it was recommended that the patient be hospitalized for continued medical management and consideration of I&D. If a CT scan revealed an abscess ≥1.7cm, then I&D with specimen culture were performed. The treatment algorithm is shown in Figure 1.

Surveys

Anonymous surveys were designed in conjunction with the institutional quality and safety department and administered to the EM residents at OhioHealth Doctors Hospital in Columbus, Ohio, for self-reported confidence levels in the management of PTA both pre- and four months post-protocol implementation. EM residents were surveyed about their confidence level in managing PTA in nine various clinical questions. For each question, the residents reported their confidence level on a 10-point Likert scale (Table 1), with 1 being not confident at all to 10 being confident all of the time. The OhioHealth Office of Human Subjects Protections issued approval 1935670-1.

**Table 1:** Self-reported confidence level and Likert scale conversion

Confidence level	1	2, 3, 4	5	6, 7, 8 ,9	10
Actual scale	Not at all confident	Slightly confident	Confident most of the time	Quite confident	Confident all of the time

Likert scale data were analyzed at the interval measurement scale. Descriptive statistics include the mean for central tendency and the standard deviation for variability. Paired t-tests compared means before and after intervention. Data analysis was conducted in Microsoft Excel 365 (Microsoft, WA, USA). Statistical analysis was performed by the Institutional Quality and Safety department.

## Results

**Figure 2:**
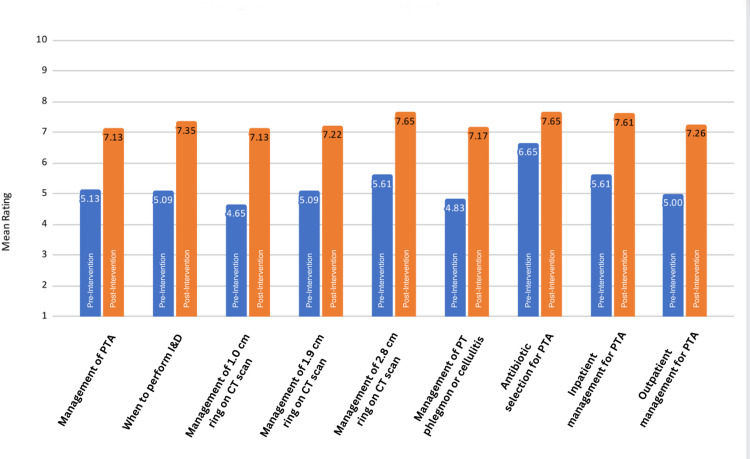
Confidence level of residents before and after intervention CT: computerized tomography, I&D: incision and drainage, PT: peritonsillar, PTA: peritonsillar abscess

**Figure 3:**
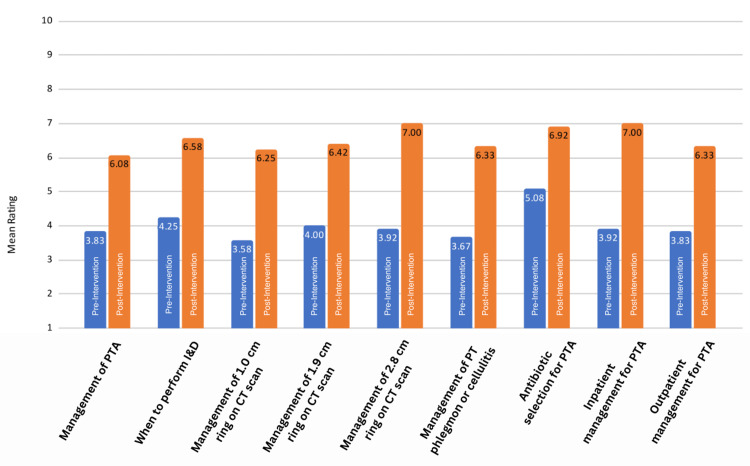
Confidence level of PGY 1 and 2 before and after intervention CT: computerized tomography, I&D: incision and drainage, PGY: post-graduate year, PT: peritonsillar, PTA: peritonsillar abscess

**Figure 4:**
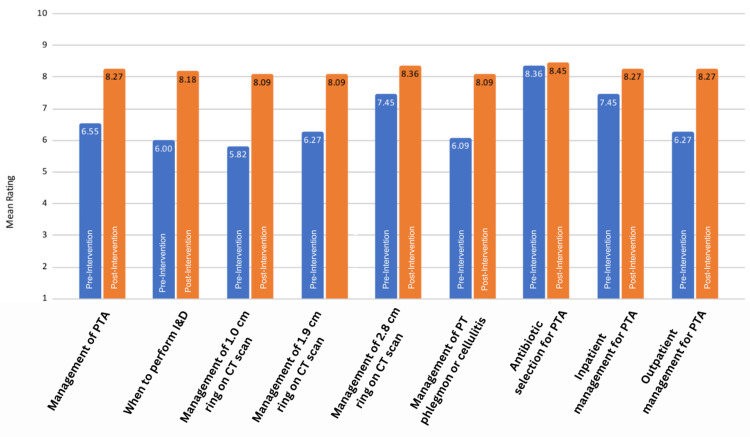
Confidence level of PGY 3 and 4 before and after intervention CT: computerized tomography, I&D: incision and drainage, PGY: post-graduate year, PT: peritonsillar, PTA: peritonsillar abscess

The study was completed over a single academic year. Twenty-seven of the 32 EM residents completed the project, giving a response rate of 84.38%. There were a total of 23 pre- and post-data sets (Figure 2), with further stratification into 12 for post-graduate year (PGY) 1 and 2 residents (Figure 3) and 11 for PGY 3 and 4 residents (Figure 4). The mean confidence level for all residents increased significantly after the implementation of the protocol (p<0.001), as shown in Table 2. The increase in confidence level for “antibiotic selection for PTA” (p=0.72) and “inpatient PTA management” (p=0.20) was not statistically significant for PGY 3 and 4 residents (Table 4). The increase in confidence level was higher overall for PGY 1 and 2 residents (95% CI 2.25 ± 1.09, p<0.001) than for PGY 3 and 4 residents (95% CI 1.73 ± 1.09, p=0.003), as shown in Table 3 and Table 4, respectively.

**Table 2:** Change in confidence level before and after intervention for all residents (n=23) CI: confident interval, CT: computerized tomography, PT: peritonsillar, PTA: peritonsillar abscess, I&D: incision and drainage

Confidence level statement	Mean rating before intervention	Mean rating after intervention	Mean rating change (95% CI)	Statistical significance (p<0.05)
Management of PTA	5.13	7.13	2.00 ± 0.69	p<0.001
When to perform I&D	5.09	7.35	2.26 ± 0.73	p<0.001
Management of 1.0 cm ring on CT scan	4.65	7.13	2.48 ± 0.81	p<0.001
Management of 1.9 cm ring on CT scan	5.09	7.22	2.13 ± 0.66	p<0.001
Management of 2.8 cm ring on CT scan	5.61	7.65	2.04 ± 0.86	p<0.001
Management of PT phlegmon/cellutilis	4.83	7.17	2.35 ± 0.71	p<0.001
Antibiotic selection for PTA	6.65	7.65	1.00 ± 0.70	p=0.007
Inpatient PTA mgmt	5.61	7.61	1.96 ± 0.98	p<0.001
Outpatient PTA mgmt	5.00	7.26	2.26 ± 0.78	p<0.001

**Table 3:** Change in confidence level before and after intervention for EM PGY 1 and 2 residents (n=12) CI: confident interval, CT: computerized tomography, PT: peritonsillar, PTA: peritonsillar abscess, EM: emergency medicine, PGY: post-graduate year, I&D: incision and drainage

Confidence level statement	Mean rating before intervention	Mean rating after intervention	Mean rating change (95% CI)	Statistical significance (p<0.05)
Management of PTA	3.83	6.08	2.25 ± 1.09	p<0.001
When to perform I&D	4.25	6.58	2.33 ± 1.28	p=0.002
Management of 1.0 cm ring on CT scan	3.58	6.25	2.67 ± 1.25	p<0.001
Management of 1.9 cm ring on CT scan	4.00	6.42	2.42 ± 1.03	p<0.001
Management of 2.8 cm ring on CT scan	3.92	7.00	3.08 ± 1.28	p<0.001
Management of PT phlegmon/cellutilis	3.67	6.33	2.67 ± 1.16	p<0.001
Antibiotic selection for PTA	5.08	6.92	1.83 ± 1.11	p=0.003
Inpatient PTA mgmt	3.92	7.00	3.08 ± 1.20	p<0.001
Outpatient PTA mgmt	3.83	6.33	2.50 ± 1.26	p<0.001

**Table 4:** Change in confidence level before and after intervention for EM PGY 3 and 4 residents (n=11) CI: confident interval, CT: computerized tomography, PT: peritonsillar, PTA: peritonsillar abscess, EM: emergency medicine, PGY: post-graduate year, I&D: incision and drainage, *not statistically significant

Confidence level statement	Mean rating before intervention	Mean rating after intervention	Mean rating change (95% CI)	Statistical significance (p<0.05)
Management of PTA	6.55	8.27	1.73 ± 1.00	p=0.003
When to perform I&D	6.00	8.18	2.18 ± 0.89	p<0.001
Management of 1.0 cm ring on CT scan	5.82	8.09	2.27 ± 1.24	p=0.002
Management of 1.9 cm ring on CT scan	6.27	8.09	1.82 ± 0.94	p=0.002
Management of 2.8 cm ring on CT scan	7.45	8.36	0.91 ± 0.82	p=0.03
Management of PT phlegmon/cellutilis	6.09	8.09	2.00 ± 0.95	p<0.001
Antibiotic selection for PTA	8.36	8.45	0.09 ± 0.56	p=0.72*
Inpatient PTA mgmt	7.45	8.27	0.82 ± 1.34	p=0.20*
Outpatient PTA mgmt	6.27	8.27	2.00 ± 1.12	p=0.002

## Discussion

The landscape for the management of PTA in the ED has benefited from ongoing research in the domains of identification and management. Evolving trends include efforts directed at reducing the use of CT scans for uncomplicated cases [7,8]. Intraoral ultrasound in the ED is increasingly being utilized as a management tool to guide interventions. This point-of-care tool has the potential to reduce the length of stay and need for admission in select patients presenting with PTA [9,10].

Despite the fact that PTA is the most common deep-space infection of the neck, management remains highly variable in the emergency department. A review of assessment and treatment variability suggests this is not isolated to the United States and demonstrates an opportunity to develop best practice patterns to guide appropriate care and minimize unnecessary testing [8,11].

The skill set learned in residency serves as the foundation for lifelong learning and care by emergency physicians. A treatment path based on best care guidelines allows for appropriate care of PTA patients and has downstream consequences in terms of resource utilization and patient care [12]. Management plans learned during residency are highly variable and may leave EM feeling ill-equipped to perform PTA, including which patients would benefit from either aspiration or I&D versus medical management [13].

Clinical pathways, when used properly, serve as an excellent tool to balance safe and efficient patient care while providing a framework for the growth and development of resident knowledge [14]. Additionally, resident involvement in quality projects is essential to their training. This is not only a requirement of accreditation for accredited programs but is also a best practice to prepare learners to participate in essential quality activities as part of a medical staff [15]. For these reasons, this protocol-based, best-practice quality improvement approach was taken to determine the feasibility of an EM residency training program.

Based on the survey findings, it can be inferred that the PTA protocol effectively supports residents in managing PTA cases. Therefore, it is recommended to integrate this protocol into the training program for EM residents, particularly those in their first and second years of residency (PGY 1 and 2). It is expected that the overall increase in confidence levels will be lower for senior residents (PGY 3 and 4) compared to the PGY 1 and 2 groups, given their fewer years of training. However, the results suggest that even senior residents can benefit from the implementation of the PTA protocol.

The increase in confidence levels that were not significant for the statements “antibiotic selection for PTA” and “inpatient PTA management” within the PGY 3 and 4 groups was likely due to the already greater than average confidence levels for those questions in the pre-protocol survey for those groups. In fact, in the pre-protocol survey, “antibiotic selection for PTA” had the highest confidence rating of all groups, with “management of a 1.0 cm ring-enhancing lesion on a CT scan” having the lowest confidence rating of all groups.

Our study had several limitations, the first of which was resident compliance with the protocol. The protocol was disseminated to EM residents, and education on the use of the protocol was given. EM residents were invited to ask questions freely as needed. However, complete compliance with the protocol was not measured. Second, the number of PTAs that presented during the four-month interventional period could not be controlled and was not recorded. Thus, a low number of PTAs presenting to the emergency department during this period may not be sufficient to assess the impact of the protocol. Additionally, it could not be ensured that there was an equal allocation of cases among the EM residents. Extending the interventional time frame would improve confidence in achieving sufficient case numbers.

## Conclusions

Implementation of a standardized protocol for the management of uncomplicated PTA is an effective tool for EM residents. The mean confidence level for all resident levels increased significantly after implementation of the protocol, showing a greater increase in overall confidence level for PGY 1 and 2 residents than that of PGY 3 and 4 residents. For the PGY 3 and 4 residents, the increase in confidence level was not statistically significant for “antibiotic selection for PTA” and “inpatient PTA management.” Overall, this research demonstrated the positive impact that a standardized protocol could have on resident decision-making regarding managing PTA. Future research will measure resident compliance with the protocol using a case review process, reviewing CT scans, reviewing data from I&D procedural codes, and measuring the impact on patient outcomes.
